# Predictors of mortality of patients newly diagnosed with clinical type 2 diabetes: a 5-year follow up study

**DOI:** 10.1186/1472-6823-10-14

**Published:** 2010-08-10

**Authors:** Niels de Fine Olivarius, Volkert Siersma, Anni BS Nielsen, Lars J Hansen, Lotte Rosenvinge, Carl Erik Mogensen

**Affiliations:** 1 The Research Unit for General Practice and Section of General Practice, Department of Public Health, University of Copenhagen, Copenhagen, Denmark; 2 Section of Biostatistics, Department of Public Health, University of Copenhagen, Copenhagen, Denmark; 3 Medical Department M, Århus Kommunehospital, Århus University Hospital, Århus, Denmark

## Abstract

**Background:**

At diabetes diagnosis major decisions about life-style changes and treatments are made based on characteristics measured shortly after diagnosis. The predictive value for mortality of these early characteristics is widely unknown. We examined the predictive value of patient characteristics measured shortly after diabetes diagnosis for 5-year all-cause and cardiovascular mortality with special reference to self-rated general health.

**Methods:**

Data were from a population-based sample of 1,323 persons newly diagnosed with clinical diabetes and aged 40 years or over. Possible predictors of mortality were investigated in Cox regression models.

**Results:**

Multivariately patients who rated their health less than excellent experienced increased all-cause and cardiovascular mortality. These end-points also increased with sedentary life-style, relatively young age at diagnosis and presence of cardiovascular disease (CVD) at diagnosis. Further predictors of all-cause mortality were male sex, low body mass index and cancer, while cardiovascular mortality increased with urinary albumin concentration.

**Conclusions:**

We found that patients who rated their health as less than excellent had increased 5-year mortality, similar to that of patients with prevalent CVD, even when biochemical, clinical and life-style variables were controlled for. This finding could motivate doctors to discuss perceptions of health with newly diagnosed diabetic patients and be attentive to patients with suboptimal health ratings. Our findings also confirm that life-style changes and optimizing treatment are particularly relevant for relatively young and inactive patients and those who already have CVD or (micro)albuminuria at the time of diabetes diagnosis.

## Background

In observational studies several characteristics of patients with type 2 diabetes have been linked to mortality, most importantly age, sex, blood glucose level [1-3], lipids [2], urinary albumin excretion [2,3], blood pressure [2], smoking [2,3] and comorbidity like cardiovascular disease (CVD) [2,3]. In the general population self-rated general health (SRH) predicts both future morbidity such as ischaemic heart disease [4] and mortality [5] independently of established risk indicators, but evidence of this is scarce for people with diabetes [3,6,7].

The findings in these studies are not unequivocal, partly because patients were examined at different stages in the natural history of diabetes [1,8]. In the Danish study, Diabetes Care in General Practice, a large group of people newly diagnosed with clinical type 2 diabetes were examined by their general practitioner for the most part within two weeks of diabetes diagnosis [9]. These patients have a considerable over-mortality compared with the general population [10,11]. In this group we investigated the contribution to 5-year all-cause and cardiovascular mortality of the characteristics found at the more or less acute state at diabetes diagnosis. In particular, we evaluated the possible independent effect of SRH on mortality.

## Methods

### Study population

In the Diabetes Care in General Practice study [9], 474 general practitioners from 311 practices agreed to include all subjects on their practice list who fulfilled the following criteria: (1) newly diagnosed diabetes based on hyperglycaemic symptoms and/or raised blood glucose values; (2) diagnosed from 1 March 1989 to 28 February 1992; and (3) age 40 years or over. The diagnosis was subsequently confirmed with a single fasting whole blood/plasma glucose ≥7.0/8.0 mmol/l measured at a major laboratory. Accordingly, 1,381 diabetic patients were included in the study, and a further 162 patients were excluded because of life-threatening somatic disease (31%), severe mental illness (31%) and unwillingness to participate (38%). Forty-six patients being treated with steroids at the time of diagnosis and 12 non-Caucasian patients were excluded from the analysis. This gave a final study population of 1,323 diabetic patients. A small number started insulin treatment within 180 days of diagnosis, so 97.6% of these patients were considered to have type 2 diabetes [9]. The Copenhagen and Frederiksberg Research Ethics Committee approved the study.

### Assessments

Immediately after diabetes diagnosis, the doctors recorded the following information about the patient: height and weight without shoes and outer garments; blood pressure and heart rate by routine methods after a 10-minute rest in a sitting position; sense of touch of cotton wool and pin prick on both feet; presence of dorsalis pedis or posterior tibial pulse on both feet; presence of patellar reflexes; history of myocardial infarction and/or stroke causing hospitalization; and amputation of any part of leg or foot before or at the time of diabetes diagnosis. The median time (interquartile range) from diagnosis until patients were examined by their general practitioner was 12 (4-28) days, 10 (4-27) days for survivors and 12 (5-36) days for non-survivors (*p *= 0.091). Retinopathy was assessed by practising ophthalmologists.

In questionnaires, patients gave information about the preceding year's leisure time physical activity in four categories as shown in the Additional File 1. The three non-sedentary categories were combined for the present analyses because of small numbers (*n *= 87 in all) in the two most extreme categories. SRH was evaluated with a single question: *in general, how would you rate your health at present? *The response categories were *excellent, good, fair, poor *and *very poor*. As above, the extreme categories, poor (*n *= 103) and very poor (*n *= 20), were combined for the present analyses. The SRH-question was answered by 97.6% (1291/1323) of the patients. The instruments for measuring physical activity and SRH were chosen because they are internationally recommended for health surveys [12,13]. Furthermore the SRH-question is recommended especially for mortality studies with a wide age range [14]. The patients were also asked about whether they lived alone, their education, and former or present cancer. These three questions and the question concerning physical activity where developed by The National Institute of Public Health in Denmark for The National Health Interview Surveys. All five questions (see Additional File 1) were tested for face and content validity through two rounds of pilot interviews preceded and followed by an expert hearing [15]. Information about smoking habits, angina pectoris, and intermittent claudication was collected with WHO-standardised London School of Hygiene questionnaires [16]. The entire patient questionnaire was pilot tested among patients with known type 2 diabetes before the study started.

### Definitions

Cardiovascular disease (CVD) was defined as history of myocardial infarction and/or history of stroke and/or angina pectoris and/or intermittent claudication and/or absent arterial pulses on both feet and/or amputation on the lower extremities. Peripheral neuropathy was defined as lack of a sense of pin prick and/or touch of cotton wool on at least one foot and/or absent patellar reflex on at least one knee. Diabetic retinopathy was defined as presence of at least one microaneurysm or further diabetic retinopathy.

### Assays

Samples for diagnostic plasma or whole-blood glucose tests were drawn after a minimum of 8 h fasting and analysed at local major laboratories (n = 72). A factor of 1.15 was used to convert whole-blood measurements to plasma glucose values. All remaining blood samples were analysed at Odense University Hospital. Serum total cholesterols were determined enzymatically with cholesterol esterase-cholesterol oxidase-peroxidase reagent and fasting serum triglycerides with a lipase-glycerolkinase-glycerol-3-phosphate oxidase-peroxidase reagent. Urinary albumin concentration was measured in a freshly voided morning urine at Århus University Hospital by a polyethylene glycol radioimmunoassay. Quality assurance was obtained with commercial control preparations.

### Cause of death

The vital status was certified and surviving patients were censored on 26 September 1995. The cause of death for 297 of 298 deceased patients was taken as the first entry on the death certificate from the electronic version of the Danish National Death Register. The cause of death was from the general practitioner in one case in which it was not recorded in the register. The coding of cause of death was validated with written information from the general practitioners and by manually going through copies of the original death certificates which could be acquired for 296 patients. An autopsy had been done in 55 (18.6%) cases. The original entries were retained because recoding of CVD as cause of death was only relevant in one patient.

Of 292 patients with a registered manner of death, 281 died a natural death, 8 died from accidents and 3 from suicide. The Danish National Death Register changed coding of cause of death from ICD-8 to ICD-10 from 1 January 1994. The following ICD-8/ICD-10 codes were used to classify cause of death: CVD, 390-458/I00-I99; ischemic heart disease, 410-414/I20-I25; and stroke, 430-438/I60-I69. Diabetes mellitus was mentioned on 131 (44.3%) of 296 death certificates.

### Statistical analysis

The influence on all-cause mortality of baseline characteristics measured at diabetes diagnosis, i.e. socio-demographic, clinical, biochemical and behavioural variables as well as complications and SRH, was investigated in Cox proportional hazard regression models. In these models the death intensity was represented as a function of patient age, multiplicatively affected by the characteristics, and patients entered the risk set at the time of diagnosis. The effect of a characteristic was assessed by a hazard ratio, i.e. the ratio of the age-specific mortality rate in a specific category of a patient characteristic compared with to the mortality rate in a reference category. We chose to use age as time scale instead of diabetes duration, which is more common, because we considered that the hazard changes more with age than with diabetes duration [17]. In such analyses, a diagnosis-age effect compares the mortality hazards of those diagnosed at an old age versus those diagnosed at a younger age at each age, and a hazard ratio of e.g. 0.5 indicates that those diagnosed at an older age have half the hazard of those diagnosed at a young age given that they have the same age (and hence different diabetes duration), i.e. the age-specific mortality hazard is lower for those diagnosed at an older age relative to those diagnosed at a young age. We used the PROC PHREG procedure from the SAS statistical package ver. 9.1.

We assessed the effects of the diagnostic characteristics both separately and in multivariate models (Table 1). In three such models the aim was to assess the effect of SRH while adjusting for possible confounding effects of other characteristics. Model I adjusted for SRH and all other baseline characteristics except chronic conditions. Model II included all diagnostic characteristics inclusive of chronic conditions. In model III, SRH was omitted from model II to examine whether SRH mediated the effect of the remaining characteristics on mortality by comparing hazard rates with those in model II. In a further analysis all interactions between a characteristic and SRH, and the interaction between age and sex, were added to model II. Akaike's Information Criterion (AIC) and Bayes' Information Criterion (BIC) were used to compare the quality of the models relative to both fit and size [18]. When variables are added to a model the model fit improves, and this improvement has to be seen relative to the increase in model size. The scores of AIC and BIC perform the trade-off, each in a slightly different way, between the model likelihood and the number of parameters in the model. The lowest value of the information criterion indicates the model of best quality. The effects of selected characteristics were illustrated in Table 2 by the median life expectancy. This was projected by model II through the 0.5-point of the survival function [19].

**Table 1:** Predictors of 5-year all-cause mortality of patients with newly diagnosed type 2 diabetes.

		Univariate analyses			Multivariate analyses				
				
		Survivors *n *= 1,025	Deceased *n *= 298		Model I ***n *= 1,108**^**h**^	**Model II**^**g**^ ***n *= 1,108**^**h**^	Model III ***n *= 1,108**^**h**^	Missing values	**SRH interactions, *p*-value**^**i**^
Information criteria, AIC/BIC					2029.7/2087.7	2004.7/2076.4	2009.6/2071.0		
Sex	Women	495 (48)	124 (42)	1	1	1	1		
	Men	530 (52)	174 (58)	1.63 (1.29-2.06)***	1.65 (1.19-2.28)**	1.63 (1.17-2.26)**	1.58 (1.14-2.20)**	0	0.96
Age at diabetes diagnosis (years)		63.0 (53.9-71.4)	72.6 (64.7-79.5)	0.92 (0.86-0.98)*	0.82 (0.75-0.89)***	0.80 (0.73-0.87)***	0.80 (0.73-0.87)***	0	0.38
Living alone	No	710 (71)	161 (56)	1	1	1	1		
	Yes	293 (29)	126 (44)	1.07 (0.83-1.38)	1.21 (0.90-1.65)	1.26 (0.93-1.71)	1.26 (0.93-1.71)	33	0.68
Education	Higher	227 (23)	45 (16)	1	1	1	1		
	Basic	749 (77)	230 (84)	1.01 (0.72-1.39)	1.11 (0.76-1.63)	1.15 (0.78-1.67)	1.17 (0.80-1.72)	72	0.54
Diagnostic plasma glucose (mmol/l)^a^		13.7 (10.7-17.0)	13.8 (10.6-17.5)	1.12 (0.80-1.57)	0.99 (0.66-1.46)	1.08 (0.73-1.61)	1.11 (0.75-1.65)	0	0.018
Fasting triglycerides (mmol/l)		1.99 (1.39-2.93)	1.89 (1.40-2.67)	1.02 (0.98-1.05)	0.97 (0.90-1.04)	0.98 (0.91-1.05)	0.98 (0.92-1.06)	25	0.98
Total cholesterol (mmol/l)		6.3 (5.5-7.2)	6.0 (5.1-6.9)	0.93 (0.85-1.01)	0.97 (0.86-1.09)	0.94 (0.83-1.05)	0.93 (0.83-1.05)	20	0.28
Urinary albumin (mg/l)^a^		11.0 (5.6-25.0)	15.9 (7.2-42.3)	1.16 (1.07-1.26)***	1.10 (0.99-1.21)	1.10 (1.00-1.21)	1.09 (0.99-1.21)	39	0.18
Body mass index (kg/m^2^)		29.4 (26.4-33.2)	28.0 (25.6-31.4)	0.98 (0.95-1.00)	0.97 (0.94-1.00)*	0.97 (0.94-1.00)*	0.97 (0.94-1.00)*	4	0.11
Resting heart rate (beats/min)^b^		76 (68-81)	76 (72-86)	1.18 (1.08-1.29)***	1.06 (0.95-1.19)	1.07 (0.95-1.19)	1.08 (0.96-1.20)	5	0.58
Systolic blood pressure (mmHg)^b^		150 (130-160)	150 (130-170)	0.95 (0.90-1.01)	1.02 (0.96-1.10)	1.05 (0.98-1.12)	1.06 (0.99-1.13)	7	0.69
Physical activity	Active	768 (77)	167 (59)	1	1	1	1		
	Sedentary	231 (23)	118 (41)	1.57 (1.23-2.01)***	1.67 (1.23-2.25)***	1.62 (1.21-2.19)**	1.59 (1.19-2.13)**	39	0.73
Smoking	Never	320 (33)	86 (30)	1	1	1	1		
	Former	319 (33)	103 (36)	1.50 (1.12-2.02)**	1.38 (0.96-1.99)	1.33 (0.91-1.92)	1.35 (0.93-1.96)		
	Current	338 (35)	94 (33)	1.71 (1.25-2.33)***	1.29 (0.87-1.90)	1.30 (0.88-1.94)	1.31 (0.88-1.95)	63	0.79
Self-rated health^c d e f^	Excellent	142 (14)	15 (5)	1	1	1			
	Good	357 (36)	80 (28)	1.30 (0.84-2.04)	2.60 (1.36-4.96)**	2.46 (1.28-4.71)**			
	Fair	423 (42)	151 (53)	1.72 (1.13-2.62)*	3.02 (1.62-5.65)***	2.51 (1.33-4.71)**			
	(Very) poor	82 (8)	41 (14)	2.43 (1.48-3.98)***	2.97 (1.46-6.07)**	2.16 (1.04-4.46)*		32	-
Cardiovascular disease	No	761 (76)	137 (49)	1		1	1		
	Yes	237 (24)	141 (51)	2.11 (1.66-2.70)***		2.08 (1.56-2.77)***	2.20 (1.66-2.91)***	47	0.036
Diabetic retinopathy	No	946 (95)	252 (94)	1		1	1		
	Yes	45 (5)	16 (6)	1.08 (0.65-1.82)		1.15 (0.65-2.01)	1.10 (0.63-1.93)	64	0.28
Peripheral neuropathy	No	834 (82)	217 (75)	1		1	1		
	Yes	178 (18)	73 (25)	1.16 (0.89-1.53)		0.90 (0.65-1.26)	0.94 (0.67-1.30)	21	0.77
Cancer (former or present)	No	987 (96)	273 (92)	1		1	1		
	Yes	38 (4)	25 (8)	1.84 (1.21-2.78)**		2.30 (1.42-3.71)***	2.30 (1.43-3.70)***	0	0.71

Values of characteristics at diabetes diagnosis are numbers (%) or medians (95%-confidence intervals). Hazard ratios (95%-confidence intervals) and *p*-values are from univariate Cox regression analyses and from 3 multivariate Cox regression analyses with and without self-rated health and/or complications at diabetes diagnosis ^g^.

^a ^The characteristic is log-transformed in the analysis

^b ^Hazard ratio estimates risk increase per 10 units of the characteristic

^c ^Information criteria for the univariate Cox regression model (*n *= 1,108 ^h^): AIC = 2162.2; BIC = 2172.5

^d ^Wald test for the overall effect of self-rated health: *p*-value = 0.007 (model I); *p*-value = 0.036 (model II)

^e ^Wald test for the equality of the three self-rated health effects: *p*-value = 0.22 (model I); *p*-value = 0.80 (model II)

^f ^Trend test including the SRH variable as a continuous variable in the multivariate regression analysis: p-value < 0.0001 (model I); p-value = 0.091 (model II)

^g ^Test for proportional hazards: a Wald test for the joint significance of the interaction between log(time) and each of the model variables: *p*-value = 0.62

^h ^All multivariate analyses are based on 1,108 entries without missing values in any of the three models; *n *for survivors/deceased = 884/224

^i ^Wald test for the interactions between each of the covariates and self-rated health when these were added to model II. The test for the interaction between age at diabetes diagnosis and sex had *p *= 0.44

* *p *< 0.05, **p ≤ 0.01, ***p ≤ 0.001

**Table 2:** Estimated life expectancy of newly diagnosed type 2 diabetic patients according to characteristics at diagnosis

	Age at diabetes diagnosis (years)					
	Men			Women		
	40	60	80	40	60	80
Life expectancy (years)	59.3	75.1	91.2	60.0	76.9	93.3

	*Estimated deviation from life expectancy (years)*					
Self-rated health						
Excellent	0	0	0	0	0	0
Good	-4.3	-2.8	-3.2	-2.1	-3.0	-4.1
Fair	-4.3	-2.8	-3.3	-2.1	-3.0	-4.1
Poor/very poor	-3.7	-2.2	-3.0	-1.2	-2.9	-3.5
Physical activity						
Active	0	0	0	0	0	0
Sedentary	-1.9	-1.3	-2.4	-0.7	-1.7	-2.1
Cardiovascular disease						
No	0	0	0	0	0	0
Yes	-2.3	-2.1	-2.7	-1.2	-2.9	-3.5

Values are median life expectancy (years) and deviations from median life expectancy (years) according to baseline characteristics calculated from Model II which includes all baseline characteristics. The other factors in the model are kept fixed at either the baseline comparison category (categorical variables) or the median baseline value for the survivors in Table 1 (continuous variables). Physical activity and SRH were related (*p *< 0.0001), but none of the examined interaction terms was statistically significant, so the estimated effects in the table are additive.

In Table 3 the effects of the diagnostic characteristics on cardiovascular mortality were studied, both in univariate and multivariate models as in Table 1. In these models, deaths from causes other than CVD were censored. In Table 4 a sub-group analysis of all-cause mortality of relatively healthy patients was presented. In an attempt to exclude the early effects of comorbidity on general mortality, deaths occurring within the first 6 months after diabetes diagnosis were excluded in a second sub-group analysis in Table 5. In Table 6 the all-cause mortality experience within 3 years of diabetes diagnosis or after this time was described. The proportional hazard assumption was tested for model II by adding the products of each covariate and log(time) to all covariates in the model. The Wald chi-squared test for all these products gives a test for proportional hazard. See footnotes of Tables 1 and 3. The analyses of effect heterogeneity in Table 6 also provided a test for proportional hazards.

**Table 3:** Predictors of 5-year cardiovascular mortality of patients with newly diagnosed type 2 diabetes.

		Univariate analyses			Multivariate analyses		
		
		Survived or died from cause other than cardiovascular disease *n *= 1,159	Died from cardiovascular disease *n *= 164		Model I ***n *= 1,108**^**h**^	**Model II**^**g**^ ***n *= 1,108**^**h**^	Model III ***n *= 1,108**^**h**^
Information criteria, AIC/BIC					1138.8/1186.7	1125.2/1184.4	1128.9/1179.7
Sex	Women	546 (47)	73 (45)	1	1	1	1
	Men	613 (53)	91 (55)	1.51 (1.10-2.08)**	1.52 (0.98-2.36)	1.39 (0.89-2.17)	1.36 (0.87-2.12)
Age at diabetes diagnosis (years)		64.0 (54.6-72.4)	74.4 (65.7-79.7)	0.96 (0.88-1.06)	0.86 (0.78-0.97)**	0.84 (0.75-0.94)**	0.84 (0.75-0.94)**
Living alone	No	787 (69)	84 (54)	1	1	1	1
	Yes	347 (31)	72 (46)	1.10 (0.78-1.54)	1.23 (0.82-1.84)	1.24 (0.83-1.86)	1.28 (0.85-1.92)
Education	Higher	246 (22)	26 (18)	1	1	1	1
	Basic	857 (78)	122 (82)	0.84 (0.54-1.29)	0.87 (0.53-1.41)	0.94 (0.58-1.54)	0.95 (0.58-1.55)
Diagnostic plasma glucose (mmol/l)^a^		13.7 (10.7-17.0)	13.6 (10.6-17.1)	1.11 (0.71-1.76)	0.90 (0.53-1.54)	1.01 (0.59-1.74)	1.03 (0.60-1.77)
Fasting triglycerides (mmol/l)		1.96 (1.38-2.91)	1.96 (1.49-2.84)	1.04 (1.01-1.07)*	0.95 (0.86-1.04)	0.95 (0.87-1.05)	0.96 (0.88-1.05)
Total cholesterol (mmol/l)		6.2 (5.4-7.1)	6.1 (5.2-7.0)	1.04 (0.94-1.14)	1.09 (0.95-1.26)	1.06 (0.92-1.23)	1.06 (0.91-1.22)
Urinary albumin (mg/l)^a^		11.1 (5.6-26.1)	19.7 (8.9-48.8)	1.24 (1.11-1.37)***	1.19 (1.05-1.36)**	1.18 (1.04-1.34)*	1.16 (1.03-1.32)*
Body mass index (kg/m^2^)		29.3 (26.3-33.0)	28.1 (25.8-31.5)	1.00 (0.97-1.03)	0.99 (0.95-1.03)	0.99 (0.95-1.03)	0.99 (0.95-1.03)
Resting heart rate (beats/min)^b^		76 (68-84)	76 (72-88)	1.17 (1.04-1.32)**	1.07 (0.92-1.24)	1.05 (0.90-1.22)	1.06 (0.92-1.23)
Systolic blood pressure (mmHg)^b^		150 (130-160)	155 (132-170)	0.97 (0.90-1.05)	1.03 (0.94-1.12)	1.04 (0.95-1.15)	1.06 (0.97-1.16)
Physical activity	Active	847 (75)	88 (57)	1	1	1	1
	Sedentary	282 (25)	67 (43)	1.63 (1.16-2.27)**	1.60 (1.07-2.40)*	1.57 (1.06-2.34)*	1.45 (0.98-2.14)
Smoking	Never	353 (32)	53 (34)	1	1	1	1
	Former	361 (33)	61 (39)	1.51 (1.03-2.21)*	1.45 (0.90-2.34)	1.47 (0.90-2.39)	1.48 (0.91-2.41)
	Current	391 (35)	41 (26)	1.28 (0.83-1.98)	1.00 (0.58-1.72)	1.02 (0.59-1.77)	1.01 (0.58-1.75)
Self-rated health^c d e f^	Excellent	152 (13)	5 (3)	1	1	1	
	Good	389 (34)	48 (31)	1.55 (0.84-2.88)	3.46 (1.35-8.87)**	3.13 (1.22-8.06)*	
	Fair	490 (43)	84 (54)	1.85 (1.03-3.33)*	3.63 (1.45-9.09)**	2.93 (1.16-7.41)*	
	(Very) poor	104 (9)	19 (12)	2.22 (1.09-4.50)*	2.47 (0.84-7.28)	1.77 (0.59-5.30)	
Cardiovascular disease	No	832 (74)	66 (44)	1		1	1
	Yes	293 (26)	85 (56)	2.58 (1.85-3.59)***		2.39 (1.62-3.51)***	2.45 (1.68-3.57)***
Diabetic retinopathy	No	1,061 (95)	137 (93)	1		1	1
	Yes	51 (5)	10 (7)	1.21 (0.63-2.33)		1.21 (0.59-2.48)	1.18 (0.58-2.40)
Peripheral neuropathy	No	933 (82)	118 (74)	1		1	1
	Yes	209 (18)	42 (26)	1.16 (0.81-1.67)		0.96 (0.62-1.47)	1.03 (0.67-1.57)
Cancer (former or present)	No	1,100 (95)	160 (98)	1		1	1
	Yes	59 (5)	4 (2)	0.49 (0.18-1.33)		0.61 (0.19-1.94)	0.59 (0.18-1.86)

Values of characteristics at diabetes diagnosis are numbers (%) or medians (95%-confidence intervals). Hazard ratios (95%-confidence intervals) and *p*-values are from univariate Cox regression analyses and from 3 multivariate Cox regression analyses with and without self-rated health and/or complications at diabetes diagnosis ^g^. Missing values are shown in Table 1.

^a ^The characteristic is log-transformed in the analysis

^b ^Hazard ratio estimates risk increase per 10 units of the characteristic

^c ^Information criteria for the univariate Cox regression model (*n *= 1,108 ^h^): AIC = 1194.4; BIC = 1203.0

^d ^Wald test for the overall effect of self-rated health: *p*-value = 0.036 (model I); *p*-value = 0.053 (model II)

^e ^Wald test for the equality of the three self-rated health effects: *p*-value = 0.80 (model I); *p*-value = 0.29 (model II)

^f ^Trend test including the SRH variable as a continuous variable in the multivariate regression analysis: p-value = 0.027 (model I); p-value = 0.55 (model II)

^g ^Test for proportional hazards: a Wald test for the joint significance of the interaction between log(time) and each of the model variables: *p*-value = 0.61

^h ^All multivariate analyses are based on 1,108 entries without missing values in any of the three models; *n *for censored/deceased from CVD = 984/124

* *p *< 0.05, **p ≤ 0.01, ***p ≤ 0.001

**Table 4:** Vital status 5 years after diabetes diagnosis and Cox models in a sub-group of healthy^a ^patients

				Hazard ratio		
				
		Survivors *n *= 595	Deceased *n *= 101	Univariate analysis	**Multivariate model II**^**b**^	**Multivariate model III**^**b**^
Sex	Women	294 (49)	39 (39)	1	1	1
	Men	301 (51)	62 (61)	1.96 (1.30-2.94)**	2.32 (1.29-4.19)**	2.23 (1.25-3.99)**
Age at diabetes diagnosis (years)		61.1 (51.8-70.0)	71.5 (62.9-77.4)	0.95 (0.85-1.08)	0.81 (0.70-0.93)**	0.81 (0.70-0.93)**
Living alone	No	421 (73)	60 (63)	1	1	1
	Yes	157 (27)	36 (38)	0.87 (0.55-1.36)	1.04 (0.57-1.90)	1.03 (0.57-1.87)
Education	Higher	139 (24)	14 (15)	1	1	1
	Basic	429 (76)	77 (85)	1.26 (0.70-2.25)	1.48 (0.73-3.02)	1.50 (0.74-3.05)
Diagnostic plasma glucose (mmol/l)^c^		13.7 (10.8-17.1)	14.7 (10.9-17.9)	1.26 (0.71-2.23)	1.15 (0.60-2.20)	1.17 (0.61-2.25)
Fasting triglycerides (mmol/l)		1.87 (1.30-2.87)	1.88 (1.37-2.83)	1.04 (1.00-1.07)*	0.99 (0.89-1.10)	1.00 (0.90-1.10)
Total cholesterol (mmol/l)		6.2 (5.5-7.1)	6.1 (5.1-7.1)	0.99 (0.88-1.13)	1.00 (0.82-1.23)	1.00 (0.81-1.22)
Urinary albumin (mg/l)^a c^		10.5 (5.3-21.7)	12.6 (7.2-28.7)	1.16 (0.97-1.40)	1.05 (0.84-1.32)	1.06 (0.85-1.32)
Body mass index (kg/m^2^)		29.1 (26.0-33.2)	28.4 (26.3-31.5)	1.00 (0.96-1.04)	1.00 (0.94-1.05)	1.00 (0.95-1.05)
Resting heart rate (beats/min)^d^		76 (68-80)	76 (72-84)	1.20 (1.01-1.42)*	1.03 (0.83-1.27)	1.02 (0.83-1.27)
Systolic blood pressure (mmHg)^d^		150 (130-160)	155 (140-170)	1.06 (0.96-1.17)	1.12 (1.00-1.26)	1.13 (1.00-1.27)*
Physical activity	Active	476 (83)	61 (64)	1	1	1
	Sedentary	100 (17)	35 (36)	1.75 (1.13-2.71)*	1.53 (0.87-2.68)	1.66 (0.97-2.86)
Smoking	Never	193 (34)	33 (34)	1	1	1
	Former	180 (32)	25 (26)	1.08 (0.64-1.83)	0.94 (0.48-1.81)	0.94 (0.49-1.82)
	Current	192 (34)	38 (40)	1.93 (1.18-3.16)**	1.58 (0.84-2.98)	1.59 (0.86-2.97)
Self-rated health ^e f^	Excellent	95 (16)	6 (6)	1	1	
	Good	229 (40)	34 (35)	1.43 (0.72-2.85)	2.16 (0.82-5.69)	
	Fair	226 (39)	47 (50)	1.69 (0.87-3.27)	2.30 (0.87-6.05)	
	Poor/Very poor	29 (5)	9 (9)	2.30 (0.94-5.61)	2.10 (0.58-7.57)	

Values of characteristics at diabetes diagnosis are numbers (%) or medians (95%-confidence intervals). Hazard ratios (95%-confidence intervals) and *p*-values are from univariate and multivariate Cox regression analyses.

^a ^This relatively healthy sub-group was defined as those patients without the following conditions at diabetes diagnosis: CVD, diabetic retinopathy, peripheral neuropathy, cancer and urinary albumin concentration ≥ 200 mg/l.

^b ^As in Table 1 and 3

^c ^The characteristic is log-transformed in the analysis

^d ^Hazard ratio estimates risk increase per 10 units of the characteristic

^e ^Wald test for the equality of the three self-rated health effects: *p*-value = 0.71 (model II)

^f ^Trend test including the SRH variable as a continuous variable in the multivariate regression analysis: p-value = 0.078 (model II)

* *p *< 0.05, **p ≤ 0.01, ***p ≤ 0.001

**Table 5:** Predictors of all-cause mortality in patients who died later than 6 months after diabetes diagnosis

		Model I	Model II	Model III
Information criteria, AIC/BIC		1934.1/1991.6	1907.5/1978.5	1911.4/1972.2
Sex	Women	1	1	1
	Men	1.66 (1.19-2.30)**	1.63 (1.16-2.28)**	1.60 (1.14-2.23)**
Age at diabetes diagnosis (years)		0.85 (0.77-0.93)***	0.82 (0.75-0.90)***	0.82 (0.75-0.90)***
Living alone	No	1	1	1
	Yes	1.23 (0.90-1.68)	1.28 (0.94-1.74)	1.28 (0.94-1.74)
Education	Higher	1	1	1
	Basic	1.12 (0.76-1.65)	1.16 (0.79-1.71)	1.18 (0.81-1.74)
Diagnostic plasma glucose (mmol/l)^a^		0.96 (0.64-1.43)	1.07 (0.71-1.59)	1.09 (0.73-1.63)
Fasting triglycerides (mmol/l)		0.97 (0.90-1.04)	0.98 (0.91-1.06)	0.98 (0.91-1.06)
Total cholesterol (mmol/l)		0.96 (0.85-1.08)	0.93 (0.82-1.04)	0.92 (0.82-1.04)
Urinary albumin (mg/l)^a^		1.09 (0.98-1.20)	1.09 (0.98-1.20)	1.08 (0.98-1.19)
Body mass index (kg/m^2^)		0.97 (0.94-1.00)*	0.97 (0.94-1.00)*	0.97 (0.94-1.00)*
Resting heart rate (beats/min)^b^		1.07 (0.96-1.20)	1.08 (0.96-1.21)	1.09 (0.97-1.21)
Systolic blood pressure (mmHg)^b^		1.04 (0.97-1.11)	1.06 (0.99-1.14)	1.07 (1.00-1.15)*
Physical activity	Active	1	1	1
	Sedentary	1.71 (1.26-2.33)***	1.66 (1.23-2.25)***	1.62 (1.20-2.17)**
Smoking	Never	1	1	1
	Former	1.38 (0.95-1.99)	1.32 (0.91-1.93)	1.35 (0.93-1.96)
	Current	1.22 (0.82-1.82)	1.25 (0.83-1.87)	1.25 (0.83-1.87)
Self-rated health^c^	Excellent	1	1	
	Good	2.60 (1.36-4.97)**	2.45 (1.28-4.70)**	
	Fair	2.87 (1.53-5.37)***	2.35 (1.25-4.43)**	
	Poor/Very poor	2.84 (1.39-5.83)**	2.03 (0.98-4.22)	
Cardiovascular disease	No		1	1
	Yes		2.17 (1.62-2.90)***	2.27 (1.71-3.00)***
Diabetic retinopathy	No		1	1
	Yes		1.21 (0.69-2.13)	1.16 (0.66-2.04)
Peripheral neuropathy	No		1	1
	Yes		0.92 (0.66-1.29)	0.95 (0.68-1.33)
Cancer (former or present)	No		1	1
	Yes		2.28 (1.40-3.73)***	2.26 (1.39-3.68)**

Values are hazard ratios (95% confidence intervals) with *p*-values from 3 multivariate Cox regression analyses with and without self-rated health and/or complications at diabetes diagnosis. Deaths occurring within 6 months of diabetes diagnosis (*n *= 25) were excluded from this analysis, which comprised 273 deceased and 1,025 survivors. These multivariate analyses are based on 1,101 entries without missing values in any of the three models; *n *for survivors/deceased = 884/217.

^a ^The characteristic is log-transformed in the analysis

^b ^Hazard ratio estimates risk increase per 10 units of the factor

^c ^Wald test for the overall effect of self-rated health: *p*-value = 0.012 (model I); *p*-value = 0.048 (model II)

* *p *< 0.05, **p ≤ 0.01, ***p ≤ 0.001

**Table 6:** Predictors of all-cause mortality in patients who died within 3 years of diabetes diagnosis or after this time

		Died within 3 years of diabetes diagnosis *n *= 153	Died 3 years after diabetes diagnosis or later *n *= 145	**Heterogeneity, *p*-value**^**c**^
Sex	Women	1	1	
	Men	1.51 (1.15-2.00)**	1.77 (1.34-2.35)***	0.30
Age at diabetes diagnosis (years)		0.88 (0.78-1.01)	0.88 (0.77-1.01)	0.50
Living alone	No	1	1	
	Yes	0.99 (0.73-1.34)	1.19 (0.86-1.64)	0.31
Education	Higher	1	1	
	Basic	0.86 (0.61-1.23)	1.20 (0.84-1.71)	0.014
Diagnostic plasma glucose (mmol/l)^a^		1.08 (0.77-1.53)	1.16 (0.83-1.64)	0.11
Fasting triglycerides (mmol/l)		1.02 (0.98-1.06)	1.02 (0.97-1.07)	0.95
Total cholesterol (mmol/l)		0.91 (0.83-0.99)*	0.94 (0.86-1.03)	0.079
Urinary albumin (mg/l)^a^		1.14 (1.04-1.24)**	1.18 (1.08-1.30)***	0.24
Body mass index (kg/m^2^)		0.97 (0.95-1.00)*	0.98 (0.96-1.01)	0.043
Resting heart rate (beats/min)^b^		1.17 (1.06-1.28)***	1.20 (1.09-1.31)***	0.081
Systolic blood pressure (mmHg)^b^		0.95 (0.90-1.00)	0.96 (0.91-1.02)	0.047
Physical activity	Active	1	1	
	Sedentary	1.29 (0.94-1.75)	1.99 (1.47-2.70)***	0.020
Smoking	Never	1	1	
	Former	1.42 (0.97-2.08)	2.05 (1.42-2.94)***	
	Current	1.36 (0.95-1.93)	1.68 (1.18-2.40)**	0.12
Self-rated health	Excellent	1	1	
	Good	1.17 (0.71-1.92)	1.48 (0.90-2.43)	
	Fair	1.59 (1.02-2.48)*	1.90 (1.21-2.99)**	
	Poor/Very poor	2.05 (1.14-3.68)*	2.96 (1.66-5.27)***	0.31
Cardiovascular disease	No	1	1	
	Yes	1.96 (1.47-2.61)***	2.35 (1.73-3.19)***	0.29
Diabetic retinopathy	No	1	1	
	Yes	0.84 (0.39-1.80)	1.41 (0.71-2.80)	0.32
Peripheral neuropathy	No	1	1	
	Yes	0.94 (0.65-1.36)	1.47 (1.04-2.10)*	0.062
Cancer (former or present)	No	1	1	
	Yes	1.51 (0.84-2.70)	2.29 (1.31-4.02)**	0.30

Values are hazard ratios (95% confidence intervals) with *p*-values from univariate Cox regression analyses with separate hazard ratios for deaths within 3 years of diabetes diagnosis and after this time. *n *= 1,025 for survivors.

^a ^The characteristic is log-transformed in the analysis

^b ^Hazard ratio estimates risk increase per 10 units of the factor

^c ^*p*-values from the Wald test for the null-hypothesis that the hazard ratios for deaths < 3 years and ≥ 3 years after diabetes diagnosis are the same

*p < 0.05, **p ≤ 0.01, ***p ≤ 0.001

## Results

Median age at diagnosis was 63.6 years for men and 67.5 years for women. After a median observation time before death or censoring of 1904 days (range 11-2400 days), 298 (22.5%) patients had died, 100 (33.6%) from ischemic heart disease, 30 (10.1%) from stroke, 34 (11.4%) from other cardiovascular morbidity, 68 (22.8%) from cancer and 66 (22.1%) from other causes. The total number of person-years in the study was 6447 (men: 3084, women: 3363).

### All-cause mortality

In univariate Cox regression models there was a trend of increasing general mortality with decreasing SRH (Table 1). In the three multivariate models in Table 1 the predictive value of urinary albumin, resting heart rate and smoking became non-significant. Patients diagnosed at a young age had a higher age-specific mortality than patients diagnosed at an older age, and male gender, low body mass index and low level of physical activity also increased the relative risk of death. SRH was still an important predictor, but in model II no longer with any trend (Table 1, footnote f), and the effects of the three non-excellent categories of SRH did not differ in both model I and II (Table 1, footnote e). As expected, the models including chronic conditions at diagnosis were better at predicting death than the model without this information. At a 5% significance level, SRH could not be removed from model II, which contained all baseline characteristics. Removing SRH from this model, however, changed the two information criteria in different directions (model III in Table 1), which could mean that the effect of SRH is less than anticipated from the hazard ratios. The results from model II were translated into estimated deviations from the estimated life expectancy in Table 2.

### Cardiovascular mortality

The analyses from Table 1 were replicated for cardiovascular mortality (Table 3) for which urinary albumin was a statistically significant predictor. SRH and physical activity both had approximately the same hazard ratios as in the multivariate models with all-cause mortality.

### Additional analyses

The question of inter-rater variability was analysed as a case of clustering. A model like model II from Table 1, where a robust sandwich estimator for the covariance matrix was used to account for clustering, gave the following hazard ratios for good, fair and poor/very poor SRH, respectively: 2.46 (1.22;4.97), p = 0.012; 2.51 (1.24;5.05), p = 0.010; and 2.16 (0.99;4.71), p = 0.053. I.e. we found no evidence that inter-observer variability has influenced the results.

As SRH may be related to renal threshold and presence of symptoms, we added urinary glucose at diagnosis (linearly, p = 0.78) and number of 16 specified symptoms at diagnosis (0, 1, 2, 3, and 4 or more symptoms, p = 0.93)[20] to model II (Table 1). This analysis gave the following hazard ratios (95%-confidence intervals) for good, fair and poor/very poor SRH, respectively: 2.42 (1.26;4.64), p = 0.0081; 2.50 (1.32;4.73), p = 0.0050; and 2.16 (1.04;4.49), p = 0.040. I. e. the association between SRH and mortality was virtually unchanged.

A sub-group analysis of relatively healthy persons was done (Table 4) as well as an analysis of deaths occurring within 3 years of diabetes diagnosis and after this time (Table 6). Hazard ratios for physical activity and SRH were not lessened when the analysis was restricted to either healthy persons or later deaths. In multivariate analyses excluding early deaths, the hazard ratios were by and large unchanged from Table 1 (Table 5). None of the interactions of the covariates with SRH added to model II in Table 1 were significant after Bonferroni correction (Table 6).

## Discussion

Our patients are likely to be representative of the general population of patients with newly diagnosed clinical, symptomatic diabetes in this age group because of the well-defined background population in each general practice, the unchanged inclusion activity during the inclusion period, and the small number of exclusions [9]. Six years after diagnosis, only 17% of the surviving patients had ever been treated at a diabetes clinic [9], and we suggest that our study reflects results obtained in the general population of type 2 diabetic patients rarely seen in secondary care. Studies including patients with known diabetes may be misleading because of selective survival of patients with a more favourable risk factor profile [8]. Furthermore, in studies including patients with known diabetes, SRH may be the result of living with restrictions and treatments. The high mortality rate in the present study must be viewed in light of the fact that patients were included with no upper age limit.

SRH is known to be associated with mortality and morbidity in the general population [4,5,21,22], while evidence of these associations is only slowly emerging for people with type 2 diabetes [3,6,7]. A large study with 2.4 years of follow-up used the EuroQol visual analogue scale (VAS) for self-rated health and found that a 10-point higher VAS score was associated with a 6% lower risk of vascular events [6]. We found that patients who rated their health as less than excellent had increased 5-year mortality, similar to that of patients with a history of CVD already at diabetes diagnosis (Table 2). The high response rate for our SRH-question (97.6%) indicates that the question was meaningful for the respondents, and it may be that the results from an ordinal scale with 5 categories are easier to use in the doctor-patient encounter than the results from a VAS scale.

What does SRH actually measure? Responders' grounds for rating their health probably represent a personal estimation of longevity [21], which may be done by accounting for current or previous physical health, symptom perception, personal resources and physical functioning [23,24] together with health behaviour [23,24], comparison with the health of age peers [24], and a knowledge of familial dispositions [21]. Until now there is only weak evidence to suggest that mental states affect clinical outcomes independently of conventional risk factors [25]. In the present analyses a clear trend of increasing mortality with decreasing SRH was observed univariately (Table 1), but after multivariate adjustment with all available possible confounders the three non-excellent SRH categories were no longer different (Table 1, Model II, footnote e). This means that in these patients newly diagnosed with diabetes the effect of SRH boils down to whether their health is considered to be excellent or not. It seems reasonable to assume that SRH carries risk information which cannot fully be uncovered by the clinical information available at diabetes diagnosis. The possibility of residual confounding, however, cannot be ruled out and it is probable that these unknown confounders to a considerable degree have a biological basis [22,26].

Physical inactivity has been found to be an independent predictor of mortality in people with known type 2 diabetes with risk ratios similar to those of our newly diagnosed patients [27]. In contrast to the situation with SRH, etiological knowledge supports a causal role of physical activity in the prediction of morbidity and mortality [28]. The physiological effect of exercise probably acts through some of the examined risk factors, but the hazard ratio for physical activity was not weakened in the multivariate models in Tables 1 and 3.

Our results on SRH and physical activity may have been influenced by heterogeneity among our patients in their susceptibility to dying, also known as frailty [29]. Undiagnosed or even unascertainable conditions may have contributed to precipitate the diabetes diagnosis, and these conditions may be associated with both poor SRH or low physical activity and high risk of death. We approached the unobserved heterogeneity in three additional analyses which did not substantially affect our results (Tables 4, 5 and 6).

Blood glucose, lipids and blood pressure have been found to predict mortality in population-based studies of people with known diabetes [1,2] but not in the present study. This could be due to measurement error and regression dilution bias [30], which is particularly relevant for biochemical and clinical variables in the physiologically disruptive non-equilibrium state of newly diagnosed clinical diabetes, as well as frailty and differences in multivariate modelling. On the other hand, our hazard ratios for blood glucose for example were on a level with those from earlier studies [1] although non-significant. Since our patient sample was established in the early 90's, screening for diabetes has been intensified. These initiatives to identify patients earlier in the natural history of diabetes have probably decreased the variability of many of the baseline variables measured in our study. There is, however, no reason to suppose that the causal patterns underlying the associations that we have identified are different nowadays.

## Conclusions

To conclude, we found that patients who rated their health as less than excellent had increased 5-year mortality, similar to that of patients with prevalent CVD, even when biochemical, clinical and life-style variables were controlled for. The patient seems to have a knowledge about own health, which cannot be explained by patient's objective health status as it is described with present-day technology [31]. This finding could motivate doctors and other health practitioners to discuss perceptions of health with newly diagnosed diabetic patients and be attentive to patients with suboptimal health ratings. Our findings also confirm that life-style changes and optimizing treatment are particularly relevant for relatively young and inactive patients and those who already have CVD or (micro)albuminuria at the time of diabetes diagnosis.

## Competing interests

The authors declare that they have no competing interests.

## Authors' contributions

NDFO conceived of and designed the study, carried out data collection, and drafted the manuscript. VS did the statistical analyses. LR reviewed the death certificates. NDFO, VS, ABSN, LJH, LR and CEM participated in the interpretation of data and in the revisions of the manuscript. All authors have read and approved the final manuscript.

## Pre-publication history

The pre-publication history for this paper can be accessed here:

http://www.biomedcentral.com/1472-6823/10/14/prepub

## Supplementary Material

Additional file 1 **The wording of selected patient questionnaires**. The wording of patient questionnaires about self-rated health, leisure time physical activity, cohabitation status, education, and cancer. Click here for file
