# Supplementary material for: Predictors of mortality of patients newly diagnosed with clinical type 2 diabetes: a 5-year follow up study
Source: BMC Endocr Disord. 2010 Aug 10;10:14. doi: 10.1186/1472-6823-10-14 (PMC2928228; doi:10.1186/1472-6823-10-14)
Supplement: Additional file 1 — The wording of selected patient questionnaires. The wording of patient questionnaires about self-rated health, leisure time physical activity, cohabitation status, education, and cancer. [file 1472-6823-10-14-S1.PDF]

Additional Material for the paper ***"Predictors of mortality of patients newly diagnosed with clinical type 2 diabetes: a 5-year follow up study"*** by Niels de Fine Olivarius, Volkert Siersma, Anni B. S. Nielsen, Lars J. Hansen, Lotte Rosenvinge, Carl Erik Mogensen

---

Patient questionnaires

Information about smoking habits, angina pectoris, and intermittent claudication was given in London School of Hygiene questionnaires. The wording of the remaining patient questionnaires is indicated below.

Self-rated health

"In general, how would you rate your health at present?

□ excellent

□ good

□ fair

□ poor

□ very poor"

Leisure time physical activity

"Looking back upon the preceding year, what would you say is the best description of your leisure time physical activity?

□ training hard and competition sports regularly and several times a week

□ recreational sports or heavy gardening at least 4 hours a week

□ walking, bicycling or other light activities at least 4 hours a week (include Sunday excursions, light gardening and bicycling/walking to work)

□ reading, watching television or other sedentary activities”

Cohabitation status

“Are you living alone?

□ yes

□ no”

Education

“Did you ever receive vocational training?

□ yes, describe the training:_______________

□ no”

Cancer

“Do you now have or have you formerly had some of the diseases and conditions mentioned below?

…

Cancer:

□ yes, I have now, describe:________________

□ yes, I have formerly had, describe:________________

□ no, never”
